# Enhancing Surgical Safety: Microbiological Air Control in Operating Theatres at University Medical Centre Maribor

**DOI:** 10.3390/diagnostics14101054

**Published:** 2024-05-19

**Authors:** Lidija Gradisnik, Gorazd Bunc, Janez Ravnik, Tomaz Velnar

**Affiliations:** 1 Medical Faculty Maribor, 2000 Maribor, Slovenia; lidija.gradisnik@um.si; 2 Department of Neurosurgery, University Medical Centre Maribor, 2000 Maribor, Slovenia; bunc.gorazd@gmail.com (G.B.); janez.ravnik@ukc-mb.si (J.R.); 3 Department of Neurosurgery, University Medical Centre Ljubljana, 1000 Ljubljana, Slovenia; 4 AMEU-ECM Maribor, 2000 Maribor, Slovenia

**Keywords:** surgical site infections, operating theatres, microbiological contamination, air control, good surgical practice

## Abstract

Background: the aim of the study was to assess microbiological air quality in operating theatres by determining the level of microbiological contamination of the air and critical surfaces using the passive air sampling method and compliance of the operating theatre staff with infection control measures. Materials and methods: The prospective study was conducted in the surgical block of the University Medical Centre Maribor. For two months continuously, ten operating theatres were assessed for microbial contamination of air and surfaces during quiet and active times of the day. A passive air sampling method with Petri dishes on an agar specially adapted for this purpose (plate count agar) was used. In addition, ten surgical procedures were observed to assess staff compliance with recommended practises. Results: Air samples met microbiological standards in all operating theatres. In both sampling sessions of the day (quiet and active periods), microbial contamination of the air was always within the limit of 10 CFU/m^3^. The average number of bacterial colonies was zero to two during quiet phases and one to four during active phases. Approximately 60% of the isolates from the operating theatres belonged mainly to the genus *Staphylococcus*: *S. epidermidis* (36% of the isolates), *S. hominis* (17.5%) and *S. haemolyticus* (5.5%). The rest were identified as *Streptococcus anginosus* (23%) and *Bacillus* sp. (18%). Pathogenic bacteria and moulds were not present. In regard to staff compliance with good surgical practise, the former varied by behaviour and function, with non-compliance in pre-operative skin preparation and operating theatre congestion being notable. The cleanliness of the environment was satisfactory. Conclusions: Microbiological air control is extremely important for the safety and success of both surgical and postoperative practises. In spite of good results obtained in the study, further improvements in surgical staff compliance with good surgical practise are essential to reduce surgical site infections.

## 1. Introduction

Hospital-acquired infections are still an important cause of patient morbidity and mortality. In surgical practise, surgical site infections can be particularly problematic. In almost all surgical procedures, these surgical site infections (SSIs) are one of the major complications in surgical patients as they can lead to treatment complications [1,2]. Statistically, SSIs are the second-most common type of healthcare-associated infection in both Europe and the United States, and it is estimated that up to 50% of SSIs can be avoided through preventive, evidence-based strategies [1,3]. Given their negative impact, it is crucial to reduce the rate of SSIs, which includes the cleaning and disinfection procedures of surgical areas and the application of appropriate behaviours. Therefore, significant efforts have been made to optimise the operating theatre environment to reduce the intraoperative bacterial contamination rate [4,5].

Ensuring a sterile environment in operating theatres is of paramount importance for successful surgical outcomes and patient safety. Controlling microbiological contamination in the air of these rooms, therefore, plays a crucial role in minimising the risk of surgical site infections (SSIs) and other complications [2,3,4,5]. Microbiological contamination in operating theatres consists mainly of airborne pathogens, such as bacteria, fungi and viruses. These microorganisms pose a significant threat as they can adhere to surgical instruments, implants and open wounds, which can lead to postoperative infections. Factors contributing to microbial contamination of the air include the presence of theatre staff, patients and visitors, as well as inadequate ventilation systems [6,7,8,9]. Maintaining high air quality standards in operating theatres is imperative for several reasons:

(I) Infection prevention: contaminated air can introduce pathogens into the surgical area, increasing the likelihood of SSIs, which can prolong hospital stays, drive up treatment costs and even lead to patient death.
(II) Surgical success: microbial contamination can compromise the effectiveness of surgical procedures, leading to suboptimal outcomes and the need for additional procedures.
(III) Regulatory compliance: Healthcare facilities are subject to strict regulations and guidelines for air quality and infection control. Compliance with these standards is essential to ensure patient safety and avoid legal consequences.

To minimise the risk of microbial contamination in operating theatres, healthcare facilities employ various strategies. Ventilation systems with high-efficiency particulate air (HEPA) filters and ultraviolet (UV) germicidal irradiation are commonly integrated into ventilation ducts and vents to effectively remove and inactivate airborne pathogens [1,10]. Positive pressure ventilation systems ensure that the air pressure in the operating theatre is higher than in the surrounding areas to prevent the infiltration of contaminated air from adjacent rooms. Strict protocols are put in place to minimise the ingress of contaminants during surgical procedures, including the use of sterile clothing, surgical drapes and thoroughly disinfected equipment. In addition, continuous monitoring of air quality parameters, such as particle and microbial counts, enables rapid identification of deviations from acceptable standards. Routine maintenance of ventilation systems and filters is also important to ensure optimal performance [2,3,4,10].

SSIs are multifactorial and complex events with many factors contributing to the development of infection. They can be patient-related, method- or procedure-related and depend on the level of microbial contamination of the operating theatre environment [11,12]. All surgical procedures harbour the potential for contamination, with most of them initiating the infection in the surgical environment [6]. Most SSIs can be triggered by the patient’s indigenous or endogenous flora or acquired during surgical procedures in the operating theatre when microorganisms enter the exposed wound, although the exact extent to which each of these factors contributes to infection is not known [1,3]. Therefore, disinfection of the surgical site and wound as well as the choice of surgical material are of crucial importance [13,14]. In addition to other infection control and prevention measures, air pollution control in operating theatres is a significant factor in patient safety. The air can be contaminated from outside, which can be prevented by filters and pressurised ventilation. Another source of contamination is the patients and staff in the operating theatre. Strict rules, therefore, apply to both patients and operating theatre staff. Clinical studies have confirmed that up to 90% of bacterial contamination of the wound after surgery comes from colony-forming units (CFUs) from the air in the operating theatre. If the number of CFUs is reduced, the number of infections also decreases [5,6,7,11].

As operating theatres are a critical environment for potential SSIs, a sterile and controlled atmosphere is required to minimise the risk of infection and ensure patient safety. Therefore, great efforts have been made to monitor and maintain optimal air quality [2,3]. Air quality in the operating theatre is subject to strict standards and guidelines established to prevent SSIs and maintain aseptic conditions. These standards generally relate to parameters such as particulate matter, microbial contamination and gas concentrations [1,15]. There are numerous methods for air sampling to prevent airborne contaminants from compromising the sterile field and increasing the risk of postoperative complications:

(I) Passive sampling methods: Airborne particles or microorganisms are collected without the use of mechanical devices. Sedimentation plates, also known as settling plates, are often used for passive sampling (Petri dishes are most commonly used for this purpose). These plates are strategically placed in the operating theatre and left for a period of time to allow particles to settle on the agar surface. Although sedimentation plates are easy to use and inexpensive, they provide limited information on particle size distribution and may underestimate microbial contamination [1,15,16,17,18].
(II) Active sampling methods: These methods use mechanical devices to actively draw air samples into collection media. Airborne particle counters are often used for real-time monitoring of particulate matter in operating theatres [18,19]. These devices use optical or light scattering techniques to quantify airborne particles based on their size and concentration. Impaction samplers, such as the Andersen sampler, utilise the principle of inertial impaction to collect airborne particles on agar plates or collection filters. These samplers offer higher sensitivity and precision compared to passive methods [18,20].
(III) General microbiological sampling methods: Microbiological air sampling methods focus on the detection and quantification of microbial contaminants in the operating room environment. Andersen samplers, which consist of multiple stages with different-sized perforations, are also commonly used for this method of microbiological sampling. These samplers allow the selective collection of airborne microorganisms based on their size and aerodynamic properties. Surface sampling methods, such as contact plates or swabs, are used to assess microbial contamination of surfaces in the operating theatre [1,18,19]. Molecular methods, including polymerase chain reaction (PCR), enable rapid detection and identification of specific microbial species and provide valuable information for infection control measures [2,3,4,16,17,18,19].
(IV) Real-time monitoring systems integrate multiple sensors and instruments to continuously monitor air quality parameters in operating theatres. These systems provide real-time data on particulate matter concentration, microbial counts, gas concentration, temperature and humidity, enabling proactive measures to maintain aseptic conditions. Modern monitoring systems may have wireless connectivity, data logging capabilities and alarm functions to alert healthcare personnel to deviations from acceptable air quality standards [21,22].
(V) Gas sampling methods: Although not used for microbiological purposes, gas sampling methods are used to monitor the concentration of gases and volatile organic compounds in operating theatres. Direct-reading gas detectors, equipped with sensors that can detect specific gases such as oxygen, carbon dioxide and volatile anaesthetics, allow real-time monitoring of gas concentrations [23]. When sampling with sorbent tubes, the gases are passively collected on an adsorbent and then analysed in a laboratory using techniques such as gas chromatography. Gas chromatography allows the separation and quantification of individual gas components, which enables the identification of potential sources of contamination and the assessment of occupational exposure [23,24].

The use of different methods for air sampling in operating theatres can provide valuable insights into their effectiveness and practical utility. Dallolio et al. used an active air sampling method with the Surface Air System Sampler, while surfaces were analysed using the contact plate method with the RODAC (Replicate Organism Direct Agar Contact) impression technique and calcium alginate swabs. None of the air samples exceeded the microbiological reference values [1]. Sampling of surfaces can be performed in two different ways, depending on whether the surface is flat, regular or irregular. The total viable count (TVC) was then determined on plate count agar (PCA) [1]. Other authors have also used similar methods, namely passive and active air samplers and PCA for the growth of microorganisms [1,3,5,6]. These reports highlight cases where air sampling has helped to identify sources of contamination, evaluate the effectiveness of infection control measures or validate the performance of air purification systems.

The aim of our study was to monitor microbiological air quality in operating theatres by determining the level of microbiological contamination of air and critical surfaces using the passive air sampling method, and to assess the methodological or procedural factors contributing to SSIs, including compliance with preventive measures by surgical staff.

## 2. Materials and Methods

### 2.1. Operating Theatre Conditions

This prospective study was conducted in the surgical block of the University Medical Centre Maribor to investigate the microbiological quality of the air and the extent of microbiological contamination of critical surfaces in the operating theatres. The operating block in our centre consists of 12 operating theatres, which were examined. The intensive care unit (ICU) was also analysed for comparison. All operating theatres have a similar organisation of adjacent rooms, similar structural features and similar admission procedures for patients and staff. All operating theatres are ventilated with a laminar airflow system that provides positive room pressure and 12 to 15 air changes per hour. The temperature is usually set in the range of 20 to 24 °C and the relative humidity set to 60%. Laminar flow technology is used to avoid turbulence, enabling a unidirectional or uniform airflow. The airflow velocity is approximately 0.45 m/s.

Cleaning of the operating theatres was carried out by competent and trained staff between each operation and at the end of the daily operating session. Between one surgery and the next, the cleaning protocol in sequence included the following:

(I) Removal and disinfection of biological material from visibly contaminated surfaces with 10% sodium hypochlorite;
(II) Removal of debris from surgical and anaesthesiological equipment and mobile operating and instrument tables;
(III) Cleaning and disinfection of all surfaces.

In accordance with generally recognised principles, surface cleaning was always carried out from the cleanest area to the dirtiest area and from the highest to the lowest level. All surfaces that come into contact with patients’ blood or body fluids (e.g., operating table, pile and equipment) and surfaces that are frequently touched by hands (e.g., operating theatre lighting, touchscreens, monitors, tables, trolleys and keypads) were treated with a solution of detergent (non-ionic tensioactive) and disinfectant (active chlorine 1080 ppm), which was allowed to act on the exposed surfaces for at least five minutes. The floor was washed with a solution of detergent and disinfectant (active chlorine 540 ppm). The same operational protocol (in terms of surface disinfection, preparation of surgical instruments, patient preparation, antibiotic prophylaxis and behavioural norms) was applied in each “resting” operating theatre.

### 2.2. Microbiological Sampling

The tests were carried out continuously for two months. Air and surface samples were taken from each operating theatre for microbiological analysis. The samples were taken twice a day at different standard locations: the air filter, operating table and sterile operating theatre section in the early morning hours when the operating theatre was quiet, and in the afternoon hours during the active phase when surgeries were in progress. Therefore, two phases of the same daily operating session were chosen:

– Initial phase or quiet phase: At the beginning of each day, before the first programmed surgery, when the operating theatres had been cleaned the previous evening. In this case, sampling was carried out during the quiet phase between 9.00 pm and 7.00 am when the operating theatres were empty and there were no patients or operating theatre staff in the operating theatres.
– Active phase: When the operation was in progress and patients and staff were in the operating theatre. Sampling took place between 9 am and 2 pm when operations normally take place.

We used a passive air sampling method with Petri dishes on an agar specially adapted for this purpose (plate count agar). We covered the operating theatre, the intensive care unit and the neurosurgical ward. For each operating theatre, an air sample was taken for microbiological analysis. Petri dishes were placed in the operating theatres 1 to 1.5 m from the floor and at least 1 m from the wall and exposed for 1 h. The Petri dishes were exposed in duplicate. For each sampling, two dishes were exposed, one for aerobic and one for anaerobic growth conditions. Sampling took place at three locations: (I) in the centre of the room, approximately 0.5 m from the operating table and 1.5 m from the floor, (II) near the air filter and (III) in the sterile area of the operating theatre. The two locations where the Petri dishes were placed were classified as critical because of the increased traffic and movement of operating theatre personnel and, therefore, a higher risk of SSI. These critical areas included one within 1 metre of the operating field and one in the sterile area of the operating theatre. In the ICU, the agars were placed near the air filter and approximately in the centre of the ICU.

We were interested in (I) the air quality in operating theatres and surgical ICUs and (II) the bacteria that colonised surfaces. At the end of sampling, the plates and swabs were stored in refrigerated containers and taken to a laboratory for microbiological analysis. The colony counts were analysed after 48 h. In addition, the bacterial colonies grown on the agar plates were also characterised.

### 2.3. Microbiological Analysis

The total viable count (TVC) was determined on PCA (Italian Biolife SDA). The PCA plates obtained were incubated at 36 °C for 48 h. All colony-forming units (CFU) on PCA were counted and subcultured. The possible presence of moulds was determined on Sabouraud Dextrose Agar (Italian Biolife, SDA). After an incubation period of 14 days at 25 °C, the moulds grown on SDA were examined macroscopically and microscopically to exclude the presence of *Aspergillus* spp. The number of colony-forming units was expressed as CFU/plate.

### 2.4. Compliance to Best Practice

In addition, an assessment of compliance with preventive measures by surgical staff was carried out. Compliance to behavioural norms was evaluated by calculating the percentage frequencies of observed behaviours. Observations of surgical field preparation conditions and staff behaviour were documented in a series of 10 surgical procedures in different specialties, including the following:

Two craniotomies;
One carpal tunnel decompression in neurosurgery;
One spinal canal stenosis laminectomy in neurosurgery;
One laparoscopic cholecystectomy;
One inguinal hernia repair;
One appendectomy;
One hip replacement surgery in orthopaedics;
One knee replacement surgery in orthopaedics;
One spinal fixation procedure in traumatology.

## 3. Results

### 3.1. Microbiological Air Quality

A total of 320 samples were taken (12 in the operating theatre and in intensive care units 1 and 2; the Petri dishes were set up twice a day). In both sampling sessions of the day (initial phase and active phase), microbial contamination of the air was always within the limit of 10 CFU/m3 set by the Slovenian national guidelines for conventional operating theatres at rest [15]. The average number of bacterial colonies was zero to two during the resting phases and one to four during the active phases, except in the traumatological operating theatres, where 60 CFU and 7 CFU were counted on the Petri dish taken during the resting phase. The number of colonies was higher in the intensive care unit. During the quiet periods 8 CFU were found and during the active periods 22 CFU were found (Table 1 and Table 2).

*Pseudomonas* spp., *S. aureus*, *Enterobacteriaceae* and *Enterococci* were never detected. The majority of the identified species, about 60% of the isolates from the operating theatres, belonged mainly to the genus *Staphylococcus*: *S. epidermidis* (36% of the isolates), *S. hominis* (17.5%) and *S. haemolyticus* (5.5%). The rest of the isolates were identified as *Streptococcus anginosus* (23%) and *Bacillus* sp. (18%). Aerobes were predominant. Isolates at the air filter and near the operating table included *S. haemolyticus* and *S. anginosus*. Bacteria isolated in the sterile parts of the operating theatres included *Bacillus* sp., *S. epidermidis* and *S. hominis*. The isolates for the intensive care unit included *S. capitis*, *S. epidermidis* (multi-resistant strain), *S. hominis*, *S. warneri* and diphteroids. No moulds were isolated (Table 3).

The locations in the operating theatre differed depending on the bacteria isolated. In the Petri dishes near the air filter and near the operating table, the bacterial species were the same: *S. haemolyticus* and *S. anginosus*. In the sterile part of the operating theatre, *Bacillus* sp., *S. epidermidis* and *S. hominis*, were found (Table 4). No difference in bacterial isolates was found in the intensive care unit. In both locations, i.e., near the air filter and in the centre of the room, the same bacterial species were confirmed, namely *S. capitis*, multi-resistant *S. epidermidis*, *S. hominis*, *S. warneri* and diphteroids.

### 3.2. Compliance to Best Practise

#### 3.2.1. Operating Conditions

The main problem with non-compliance was occasional overcrowding in the operating theatre, largely due to the entry of non-essential personnel. The number of people present in the operating theatre at any one time varied between six and ten (with a mean of seven), which was mainly due to students frequently being involved in clinical practise and participating in surgical procedures. This was most prevalent during peak times between 11 am and 3 pm. There was also occasional conspicuous traffic flow caused by people unrelated to the surgery, including staff working in the operating theatre. Another area of non-compliance was the improper performance of pre-operative skin antisepsis, which was observed only occasionally. In four out of 10 operations, this procedure was performed without allowing the antiseptic solution to dry spontaneously, and it was then dried with a sterile cloth. In addition, a non-centrifugal technique was occasionally used to disinfect the surgical field and the surgical field was dried with sterile tissue.

#### 3.2.2. Personnel

The surgical staff consisted of surgeons, anaesthetists, scrub nurses, theatre nurses and occasionally pathologists. The non-operative staff consisted of anaesthetists and nurses from neighbouring operating theatres who wanted to obtain information, as well as nurses in training and doctors. All staff observed, both theatre staff and those not directly associated with surgery, adhered to theatre-specific dress and all used caps properly. Masks were not worn correctly in 7% of cases. Surgeons and theatre nurses were the most compliant with the dress code, with a non-compliance rate of 2%. Non-surgical staff were less likely to follow the rules in 21% of cases. Regarding hand hygiene and sterile gowning, which is mandatory for surgeons and theatre nurses, all surgical staff adhered to the practises. Surgical hand washing was carried out with a chlorhexidine soap solution and disinfection with 70% ethanol. Hands were dried after washing and before disinfection.

## 4. Discussion

### 4.1. Microbiological Air Quality

Surgical procedures are complex and delicate activities that require a sterile and controlled environment to minimise the risk of infection and resulting complications [1,2,12]. The air in operating theatres plays an important role in infections and serves as a reservoir for microorganisms that colonise the sterile surgical field. In a controlled environment such as an operating theatre, regular microbiological air quality monitoring is important to measure air quality. Airborne microorganisms pose a significant threat to patients undergoing surgery and increase the likelihood of postoperative infections and adverse outcomes [10,11,12,25,26]. Deep infections continue to be a major complication, leading to increased morbidity and mortality and driving up treatment costs. Infection can lead to various complications, including pain, discomfort, prolonged hospitalisation, increased risk of readmission, additional surgical procedures, intensive care, prolonged disability and escalating hospital costs [1,3,25]. The University Medical Centre Maribor has recognised this challenge and has taken comprehensive measures to improve microbiological air pollution control in its operating theatres. The University Medical Centre Maribor is a leading healthcare institution known for its commitment to excellent patient care and clinical outcomes. Therefore, the aim of our study was to monitor microbiological air quality in operating theatres using the passive method of air sampling and to evaluate the compliance of operating theatre staff with preventive measures.

As expected, the highest number of bacterial colonies was observed during peak hours when operations are taking place and operating theatres are fully occupied. At this time, the highest colony count of four was observed in one of the two trauma operating theatres. A high number of bacterial colonies (60 colonies) was also observed in the trauma operating theatre during the quiet period. However, we suspect that this was not a true result for two reasons. There is usually no staff in the operating theatre during the resting phase, and the agar examined was later damaged. We, therefore, suspect that something fell onto this agar plate and contaminated it. In another traumatological operating theatre, seven colonies were discovered during the resting phase. This value is also unusually high compared to other operating theatres but is still within the national limits for airborne colonies in operating theatres. The number of bacterial colonies was higher during active periods than during quiet periods, which is probably due to the spread of microbes by staff and the stirring up of dust from objects. With regard to the data collected, we can say that air pollution was well below the limits and the operating environment was safe during both busy and quiet periods. On the other hand, the number of colonies was higher in the intensive care unit. In the ICU, the number of colonies was higher (8 CFU during rest periods and 22 during active periods). This is understandable as traffic in the ICU is much heavier compared to that in operating theatres. In addition, patients in the ICU need constant care and observation and there are no real quiet times.

The most suitable sterile airflow in operating theatres is a sliding, vertical ceiling system that generates a laminar airflow at a velocity of 0.3 to 0.5 m/s [2,12]. In a conventional operating theatre, most orthopaedic intraoperative wound contamination is airborne. Surface contamination was significantly reduced in samples taken after the operating theatres had been prepared for subsequent procedures, suggesting that the prescribed cleaning and disinfection procedures between operations, when performed correctly, effectively reduce contamination to levels even below those observed at the beginning of the day [4,25,26]. Consequently, additional cleaning measures were carried out in the morning before the sessions to further minimise contamination levels, in line with the recommendations of certain guidelines.

The operating theatre environment is susceptible to contamination from various sources, including operating theatre staff, equipment and ambient air. To minimise this risk in our hospital, a multifaceted approach to microbiological air pollution control has been implemented, including both proactive measures and real-time monitoring systems [11,12,27]. Preventative measures have also been put in place to limit the number of colonies in the ICU. These include the following:

(I) Advanced air filtration systems: At the centre of the microbiological air purification strategy is the use of state-of-the-art air filtration systems in the operating theatres. These systems utilise HEPA (High-Efficiency Particle Air) filters to remove particles and microorganisms from the air, creating a sterile and conducive environment for surgical procedures. By continuously circulating and purifying the air, these filter systems minimise the presence of contaminants and reduce the risk of infection in surgical site operating theatres.
(II) Strict cleaning protocols: In addition to filtration systems, University Medical Centre Maribor applies strict cleaning protocols to ensure the cleanliness of operating theatres. The dedicated cleaning staff adhere to strict guidelines for disinfection and hygiene, ensuring that all surfaces, equipment and air ducts are free from microbial contamination. Regular audits and inspections ensure that cleanliness standards are maintained and promote a culture of accountability and excellent surgical hygiene.
(III) Real-time monitoring and control: To gain real-time insight into air quality and levels of microbial contamination, University Medical Centre Maribor utilises advanced monitoring systems. These systems utilise sensors and data analysis to continuously assess the number of particles in the air, microbial concentrations and other relevant parameters. By monitoring trends and deviations from baseline values, surgical teams can immediately recognise potential risks and take proactive measures to mitigate them, improving patient safety and procedure outcomes [3,25,26,27,28,29,30].

By maintaining a sterile surgical environment, we have significantly reduced the incidence of surgical site infections and associated complications. In addition, improved surgical safety has contributed to shorter hospital stays, faster recovery times and overall higher patient satisfaction. These results underline the importance of prioritising microbiological air control as a fundamental aspect of modern surgical practise [1,28,29].

Preventing surgical site infections is a complicated endeavour [7,25]. All activities directly or indirectly related to the surgical procedure contribute to infection prevention. There are numerous aspects that can be categorised into four interrelated areas: (I) environmental aspects, including the technical solution and architecture of operating theatres and ventilation systems, filters, air treatment systems and mechanical requirements; (II) reliable support services, including sterilisation and cleaning procedures, use of barrier materials, correct clothing and laundry practises, correct handling and processing of surgical materials and biomaterials, and ensuring the efficiency and personal hygiene of operating theatre and technical staff and compliance with good practise; (III) surgeons and theatre staff, surgical techniques and adherence to aseptic, anatomical and physiological principles; (IV) education and communication, including the operation of infection control, evaluation of methods in daily practise, effective recording of breaches and emphasis on good clinical practise. All of these components are almost equally important for effective infection control in surgical facilities [1,6,31,32,33].

### 4.2. Compliance to Best Practise

In terms of staff behaviour, adherence to postoperative infection prevention measures varied according to the specific measure observed and job role. We observed that antibiotic prophylaxis and surgical team preparation, such as hand washing and wearing sterile gowns for surgeons and theatre nurses, were highly adhered to. These practises appeared to be well established in all operating theatres and in the intensive care unit. Overall, surgeons and scrub nurses showed higher compliance than other operating theatre staff.

However, we noted some deviations from recommended practises in pre-operative skin antisepsis and operating theatre overcrowding. Non-compliance with pre-operative skin antisepsis often involved the use of excessive amounts of antiseptic applied using a non-centrifugal technique, contrary to the manufacturer’s instructions. Overcrowding in the operating theatre due to the presence of non-essential staff, particularly medical students, led to contamination of the environment and increased the risk of errors and distractions during surgery. This also compromised the effectiveness of the ventilation system in controlling contamination. Remarkable compliance was observed in the hand hygiene of anaesthetists, which was excellent and correctly performed in all observations.

The operations monitored in this study are part of the regional SSI surveillance plan, which aims to assess the incidence and distribution of infections in surgical facilities in the region. Although no infections were detected in the ten surgical procedures analysed in this study, no definitive conclusions can be drawn due to the limited number of procedures monitored. Our results are in concordance with other authors, reporting similar observations [33,34,35].

### 4.3. Laminar Airflow System in Our Operating Theatres

Laminar airflow ventilation systems are essential for maintaining sterile conditions in the operating theatre. In our operating theatres, they enable an ultra-clean zone around the operating area. They utilise integrated microbial sedimentation plates that create a continuous flow of microorganism-free air by reducing infectious microbes, improving air quality in critical areas [36,37]. In the operating theatres of the University Medical Centre Maribor, there are some basic requirements for air conditioning systems, including the possibility of regulating temperature and humidity. To prevent contamination, a high standard of air filtration is ensured, which is achieved by HEPA filters. These filters have a filtration rate of 99.9% and eliminate particles down to less than 1 µm. To avoid turbulence, a unidirectional or uniform flow is ensured, known as laminar flow technology.

The laminar airflow in our theatres is not measured. The strength of the ventilation can be adjusted as required using a rotary knob that regulates the opening of the air outlet. Normally, the ventilation system is always switched on and the ventilation strength is not changed. Therefore, we can say that ventilation in all operating theatres was constant and did not change. In our study, the airflow was not measured. We exposed the Petri dishes to different surfaces in the operating theatres and left them so that particles from the air settled on the agar plates. We used a passive air sampling method with Petri dishes on an agar specially adapted for this purpose (PCA).

### 4.4. Standards and Values for Microbiological Indicators

The Italian guidelines of the ISPESL (Istituto Superiore Prevenzione e Sicurezza del Lavoro) for operating theatres allow up to 5 CFU/Petri dish during quiet times and up to 25 CFU/Petri dish during peak hours [1]. The French guidelines of 1999 and 2016 [38] are similar. On this basis, the expected value of CFUs in operating theatres should not exceed 5, while values >5 and ≤15 CFU/plate are considered acceptable. Values of more than 15 on more than one surface sampled in the same operating theatre indicate hygiene deficiencies and a high risk of infection. The Slovenian national guidelines allow up to 10 CFU/Petri dish [39,40]. The results of our study show that the operating table, the air filter side and the sterile part of the operating theatre were better than the accepted standards in both sampling periods. Aerobes are the main cause of infections in neurosurgical patients in the postoperative period and the bacteria isolated from the wound were in most cases the same as those isolated in Petri dishes. Strict sterile conditions and air quality control are mandatory in operating theatres to avoid intraoperative infections [27,31,35].

The results obtained in our study fulfil all these guidelines. The microbiological status of the air and surfaces in the operating theatres of our hospital was satisfactory during rest and activity phases. None of the samples exceeded the total bacterial count specified in the national reference standards. In particular, potentially pathogenic bacteria such as *S. aureus*, *Enterobacteriaceae*, *Pseudomonas* spp. and *Aspergillus* spp., which must always be absent, were never detected on surfaces or in the air [1]. These results indicate the effectiveness of the cleaning and disinfection protocols used and the favourable characteristics of the ventilation systems, which are crucial for maintaining low levels of microbial contamination in operating theatres.

Comparable studies have of course been carried out in countries around the world. In this article, we presented our experiences on this topic, with the aim of conducting more such regular studies at a national level. We believe that this is an essential issue and that air monitoring in particular is often not sufficiently or not adequately recognised in clinical practise. We, therefore, emphasise the importance of regularly monitoring surgical safety in operating theatres and staff compliance with good practise.

### 4.5. Limitations of the Study

When conducting air sampling in operating theatres, several factors should be considered to ensure accurate and reliable results [41,42]. First, the location and placement of sampling devices should be carefully selected to collect representative air samples close to the surgical site and potential sources of contamination. We decided on three locations that we thought would be the most representative, depending on the amount of airflow and traffic in the operating theatre. Of course, the more locations we choose, the higher the chance of getting more accurate results. Other locations of interest may include the operating table in close proximity to the operating theatre area, the scrub nurse’s table and the instrument area. Sometimes so-called bedside surgical procedures are performed on the ward or in the intensive care unit. It would be beneficial to know the air pollution near the surgical site, which could have an impact on the future approach in such cases. In addition, it would be interesting to extend the study to other departments, e.g., wards, outpatient clinics, emergency departments, dressing wards (where the majority of wound care takes place) and high dependency wards. Secondly, the frequency of sampling should be determined based on the acuity of surgical procedures, patient risk factors and regulatory requirements. Compliance with relevant standards and guidelines, e.g., from regulatory bodies or professional organisations, is essential to ensure the validity and comparability of air sampling data [1,36]. With more frequent sampling, results could be different and the TVC could be higher, especially during the active phase of the day. Third, we used a passive air sampling method with sedimentation on Petri dishes instead of more sophisticated and modern methods. One reason for this was that this method was easy to obtain and relatively uncomplicated. In addition, the study was preliminary and we wanted to obtain basic results for further research. Active air sampling methods (including centrifugal samplers, membrane filtration, and cascade and impaction samplers) and surface sampling can be used for further studies [37,41,42].

### 4.6. Challenges and Future Directions

Despite significant advances in air sampling technology, several challenges remain in the area of air quality management in the operating theatre. These challenges include the dynamic nature of air pollutants, the influence of environmental factors on air quality and the need for continuous optimisation of sampling strategies [1,37,41]. Future directions in air sampling could include the development of integrated monitoring systems capable of multi-parameter analysis, the application of artificial intelligence and machine learning algorithms for data interpretation, and the exploration of novel materials and sensor technologies for improved sensitivity and selectivity [21,22,37]. We also propose further studies in our operating theatres that include different sampling locations and sampling methods, more frequent sampling, and improved growth conditions and media for slow-growing bacteria.

## 5. Conclusions

Microbiological air control plays a crucial role in the safety and success of surgical procedures. A wide range of air sampling methods is available to assess microbial contamination in the operating theatre environment. By understanding the principles, applications, advantages and limitations of these methods, healthcare facilities can implement comprehensive air quality monitoring programs tailored to their specific needs and regulatory requirements. The results of this study highlight the importance of monitoring, both in terms of environmental measures and, in particular, behavioural measures, where some critical areas have emerged. University Medical Centre Maribor is an example of a proactive approach in implementing comprehensive strategies to maintain sterile surgical environments. By utilising advanced technologies, strict protocols and continuous monitoring, our hospital has raised the standards of surgical safety and set a benchmark for excellence in patient care. As healthcare continues to evolve, investment in microbiological air purification will continue to be critical to optimise surgical outcomes and improve the overall quality of care.

## Figures and Tables

**Table 1:** The average number of bacterial colonies during the quiet periods.

Theatre	A1	A2	U	Tr1	Tr2	Ort1	Ort2	C1	C2	N	V	Th	ICU
Colonies	0	0	2	60	7	4	1	0	0	1	0	1	8

A: abdominal operation theatre, U: urology, Tr: trauma, Ort: orthopaedics, C: cardiovascular, N: neurosurgery, V: vascular, Th: thoracic surgery, ICU: intensive care unit.

**Table 2:** The average number of bacterial colonies during the active periods.

Theatre	A1	A2	U	Tr1	Tr2	Ort1	Ort2	C1	C2	N	V	Th	ICU
Colonies	1	2	1	4	2	0	1	0	1	2	1	1	22

A: abdominal operation theatre, U: urology, Tr: trauma, Ort: orthopaedics, C: cardiovascular, N: neurosurgery, V: vascular, Th: thoracic surgery, ICU: intensive care unit.

**Table 3:** Isolated bacteria species in the operating theatres and in the ICU.

Location	Bacteria
Operating theatres	*Bacillus* sp., *S. epidermidis*, *S. hominis* (sterile part of the operating theatre) *S. haemolyticus*, *S. anginosus* (air filter and the centre of the theatre)
ICU	*S. epidermidis*, *S. capitis*, *S. hominis*, *S. warneri*, diphteroids

**Table 4:** Bacteria species from various locations in the operating theatres.

Location	Bacteria Species
Sterile part of the theatre	*Bacillus* sp., *S. epidermidis*, *S. hominis*.
Air filter/1 m from the operating table	*S. haemolyticus* (MR), *S. anginosus*

## Data Availability

Data are contained within the article.
